# Significant Associations Between Blood Cell Counts and Plasma Cytokines, Chemokines, and Growth Factors

**DOI:** 10.3390/ijms26094065

**Published:** 2025-04-25

**Authors:** Lars B. Eriksson, Mats B. Eriksson, Torsten Gordh, Anders O. Larsson

**Affiliations:** 1 Department of Surgical Sciences, Uppsala University, Uppsala University Hospital, SE-751 85 Uppsala, Sweden; lars.b.eriksson@regiondalarna.se (L.B.E.); torsten.gordh@uu.se (T.G.); 2 NOVA Medical School, New University of Lisbon, 1099-085 Lisbon, Portugal; 3 Department of Medical Sciences, Uppsala University, Uppsala University Hospital, SE-751 85 Uppsala, Sweden; anders.larsson@akademiska.se

**Keywords:** blood cell count, chemokines, cytokines, intercytokine associations, plasma

## Abstract

The cytokine network plays a crucial role in regulating immune responses and facilitating intercellular communication. Cytokines are essential in numerous physiological and pathological processes. This study aimed to investigate associations between blood cell counts and a broad range of cytokines, chemokines, and growth factors. We included one hundred and sixty-five essentially healthy individuals in this study, which was approved by the Swedish Ethical Review Authority (Dnr 2015/378) and registered in EudraCT (2014-004235-39). Blood samples were collected for blood cell counts and analysis of cytokines, chemokines, and growth factors using the Proseek Multiplex Inflammation kit, Olink Bioscience, Uppsala, Sweden. Correlations between the different markers were calculated using Spearman rank correlations, adjusted for multiplicity and for multiple testing, with a significance threshold of *p* < 0.05. There were significant associations between platelet count and 23 cytokines, between white blood cell count and 8 cytokines, and between erythrocyte volume fractions and 19 cytokines. IL-6 had a central role within the cytokine network associated with platelets and was also associated with white blood cells and neutrophil cells. These findings emphasize the integrated nature of immune and hematological responses, where blood cell parameters are associated with systemic cytokine activity. The observed intercytokine associations, including cross-family interactions, may help to highlight regulatory pathways, providing potential targets for biomarker development and therapeutic intervention in immune-mediated conditions.

## 1. Introduction

Cytokines are signaling proteins secreted primarily by immune cells such as macrophages, T cells, dendritic cells, and other cell types in response to stimuli. These proteins play a critical role in regulating blood cell development, activation, and communication. The interaction between cytokines and blood cells is a cornerstone of immune system regulation and coordination. The interplay between blood cells and cytokine levels is an area of active research in immunology and systemic health. These associations provide insights into the interactions between the cytokine system and blood cells. The local and systemic inflammatory processes lead to cell activation, but the interactions between cytokines and blood cell counts are two-way communications [1,2,3].

High levels of white blood cells are often frequently associated with increased levels of pro-inflammatory cytokines (e.g., IL-1β, IL-6, TNF-α) in the blood [4,5]. This correlation indicates a systemic inflammatory response, where pro- and anti-inflammatory cytokines interrelate with a complex impact on the immune system and eventually cause multiple organ effects [6]. Elevated neutrophil counts, often seen in bacterial infections, can correspond to higher levels of cytokines, which attract more neutrophils to the site of infection or inflammation [7,8]. Neutrophil cells possess the ability to form neutrophil extracellular traps, which are released extracellularly and enable neutrophils to help counteract not only bacteria but also several other microbes, playing a crucial role in immune defense. However, excessive neutrophil extracellular entrapment may drive a massive inflammatory response and contribute to morbidity and mortality in many infectious diseases. Neutrophil extracellular traps also occur in noninfectious conditions and function as inflammatory mediators by degrading proinflammatory cytokines and chemokines [9,10,11].

Changes in lymphocyte counts, particularly T cells, can influence the levels of regulatory cytokines [12], which help to maintain immune balance and prevent excessive inflammation. Monocytes, which differentiate into macrophages and dendritic cells, are associated with cytokines like IL-1β, IL-6, and TNF-α [13]. Higher monocyte counts can lead to elevated levels of these cytokines, reflecting an active immune surveillance and response [14,15].

Even though leukocytes are the main type of cells associated with the cytokine network, erythrocytes are involved in both cytokine expression and signaling [16,17], as are platelets, which store and release a large number of both cytokines and chemokines [18,19,20]. Erythrocytes are involved in a multitude of actions, and apart from being oxygen carriers, erythrocytes may also act as cytokine reservoirs, enabling crosstalk with the immune system. Mature erythrocytes lack both nuclei and membrane-bound organelles and are thought to rely on their finite proteome throughout their ~120-day circulatory lifespan. Still, multiple lines for translation occur in mature erythrocytes, e.g., globin mRNAs are translated in mature erythrocytes, which are required for the maintenance of a normal level of globin proteins [21,22].

Cytokine families, their interactions, and their relations are crucial for the understanding of both physiological and pathophysiological reactions and events [23,24,25].

Systemic conditions such as diabetes, cardiovascular diseases, and autoimmune disorders often display altered blood cell counts and increased pro-inflammatory cytokine levels. For example, patients with diabetes may have elevated blood neutrophils and higher levels of IL-6 and TNF-α in saliva, indicating both systemic and local inflammation [26,27,28,29]. Psychological stress can also alter both blood cell counts and cytokine levels. Stress-induced leukocytosis can be accompanied by elevated levels of stress-related cytokines (e.g., IL-6) [30,31].

In summary, blood cell counts and plasma cytokine levels are interrelated, with changes in systemic immune parameters often mirrored by local cytokine production. These associations help in understanding the interplay between systemic and local immune responses and may be used in the diagnosis and monitoring of various health conditions. Although cytokines are a very crucial biological network, there is still a lack of understanding of the interactions between blood cells and the cytokine network. An important step in understanding the role of these biological mediators is to simultaneously study several cytokines to achieve a broader picture of the cytokine network rather than characterize individual cytokine responses. The aim of this study was to investigate the association between a broad range of cytokines, chemokines, growth factors, and enzymes in plasma with blood cell counts.

## 2. Results

### 2.1. Patient Characteristics

The cohort consisted of 165 individuals (53 males) with complete blood cell counts and PEA data. Mean age was 29 years, and interquartile range was 23–35 years.

The basic characteristics of the study cohort are presented in Table 1.

Numeric values for correlations and *p*-values between cytokines and related blood cells and blood cell markers are seen in Table 2. Analyses were performed by a proximity extension assay (PEA) (see Section 3 and Section 4.4).

The cytokines, chemokines, and growth factors analyzed by the Proseek Multiplex Inflammation kit are seen in Table S1.

Table S2 presents the correlations between measured cytokines and clinical variables. Statistically significant correlations were subsequently analyzed using multivariate adjustment (see Section 4.5).

### 2.2. Correlations Between Platelet Count and Cytokine and Chemokine Levels

**Figure 1:**
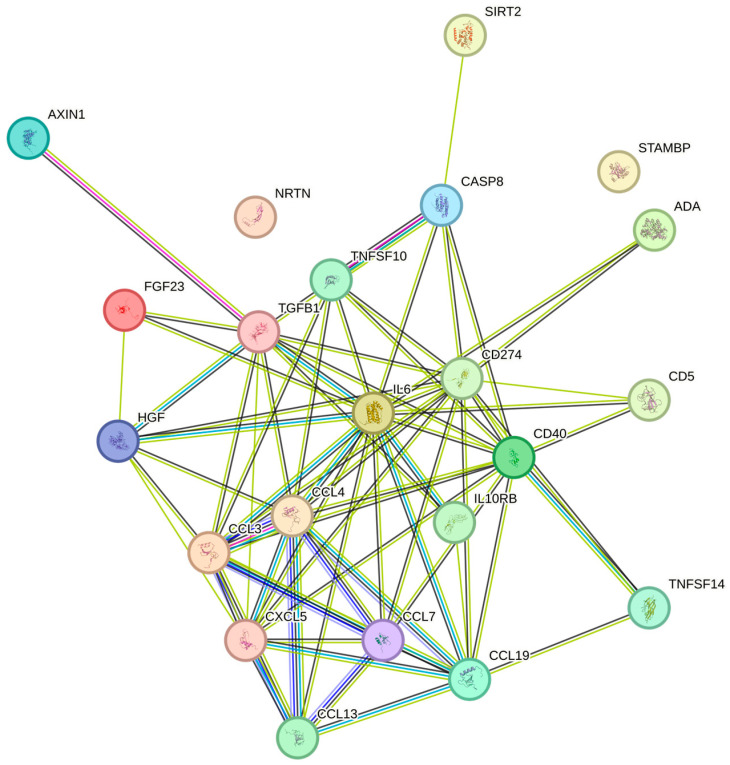
The figure represents a network of interactions among cytokines, demonstrating significant associations between circulating cytokine levels and platelets. The nodes (cytokines) are connected by edges of varying thickness, indicating interaction strength.

The platelet counts were significantly associated with FGF-23, IL-10RB, LAP TGF-beta-1, CCL19, SIRT2, IL6, VEGFA, AXIN1, CASP-8, CD40, PD-L1, CXCL5, MCP-3, STAMBP, CCL3, NRTN, HGF, CD5, TRAIL, CCL4, ADA. TNFSF14, and MCP-4 in increasing the Benjamini–Hochberg *p*-values order (Table 2). In the String plot for the cytokines that were significantly related to platelet counts, IL6 had a central role (Figure 1). The strongest associations with the platelet count were observed for MCP-3 and STAMBP (*p* = 0.052, both) and for CCL3 (*p* = 0.054), respectively.

### 2.3. Correlations Between White Blood Cell Count and Cytokine Levels

**Figure 2:**
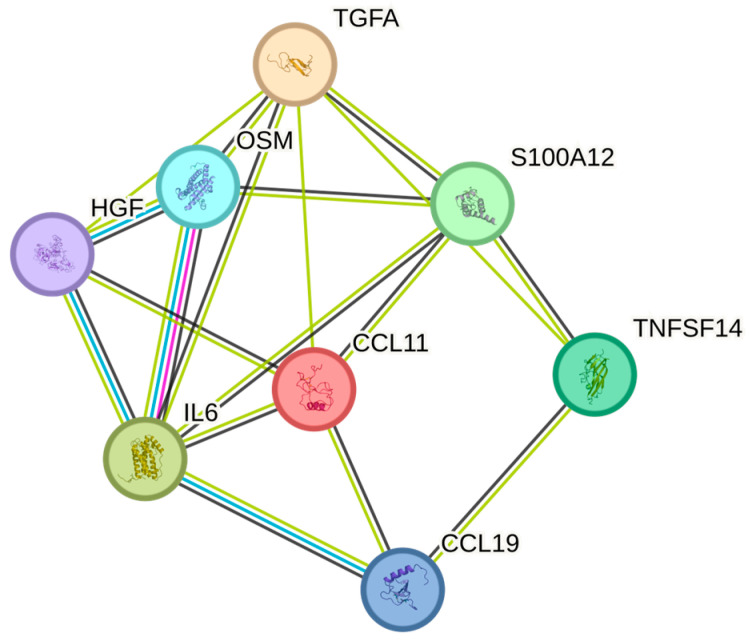
The figure represents a network of interactions among cytokines, demonstrating significant associations between circulating cytokine levels and white blood cells. The nodes (cytokines) are connected by edges of varying thickness, indicating interaction strength.

The white blood cell counts were significantly associated with OSM, IL6, HGF, TNFSF14, EN-RAGE, TGF-alpha, CCL19, and CCL11in order of increasing *p*-values (Table 2). The greatest number of associations in the String plot was between OSM and IL6 (Figure 2). The strongest associations with the WBC were observed for OSM (*p* = 1.28 × 10^−7^), IL-6 (*p* = 0.002), and HGF (*p* = 0.017), respectively.

We included both the white blood cell count and the neutrophil cell count, as they are important clinical biomarkers. The white blood cell count comprised eight significant associations with cytokines, whereas the neutrophil count was associated with six out of these cytokines. TGF-alpha and CCL11 were not associated with the neutrophil count; otherwise, the associations that were found between both the white blood cell count and the neutrophil count, respectively, were in the same magnitude.

**Figure 3:**
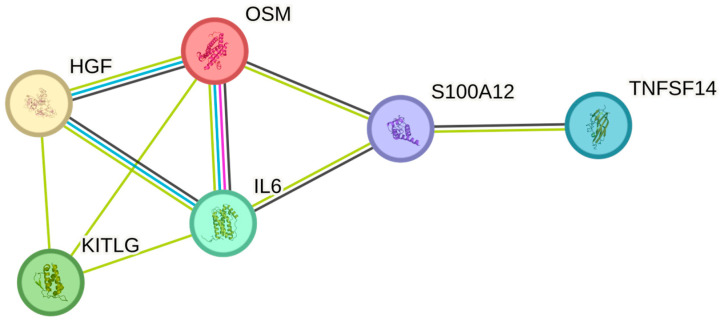
The figure represents a network of interactions among cytokines, demonstrating significant associations between circulating cytokine levels and neutrophils. The nodes (cytokines) are connected by edges of varying thickness, indicating interaction strength.

The neutrophil count showed similar associations as white blood cell counts and were significantly associated with OSM, IL6, TNFSF14, HGF, EN-RAGE, and SCF (Table 2). Similar to white blood cells, the most associations in the String plot were observed between OSM and IL6 (Figure 3). The strongest associations with the neutrophil cell count were observed for OSM (*p* = 4.73 × 10^−9^), IL-6 (*p* = 0.002), and TNFSF14 (*p* = 0.040), respectively.

### 2.4. Correlations Between Cytokine Levels and Red Blood Cell Count

**Figure 4:**
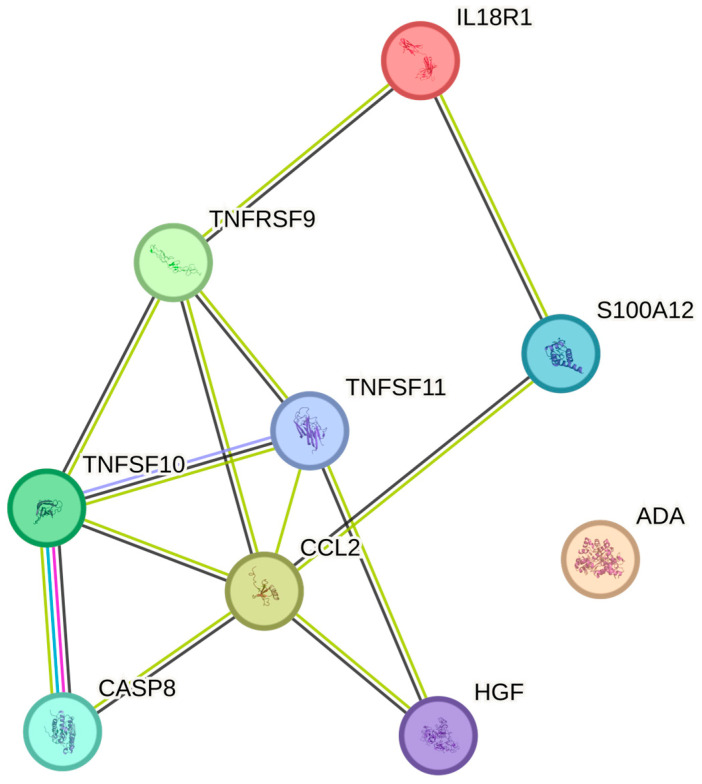
The figure represents a network of interactions among cytokines, demonstrating significant associations between circulating cytokine levels and the erythrocyte count. The nodes (cytokines) are connected by edges of varying thickness, indicating interaction strength.

The red blood cell counts were significantly associated with TRAIL, IL-18R1, HGF, MCP-1, TRANCE, TNFRSF9, ADA, EN-RAGE, and CASP-8 (Table 2). The greatest number of associations in the String plot was noted between TNFSF10 and Casp-8 (Figure 4). The strongest associations with the red blood cell count were observed for TRAIL, IL-18R1, and HGF, respectively (*p* = 0.017, all).

### 2.5. Correlations Between Cytokine Levels and the Erythrocyte Volume Fraction

**Figure 5:**
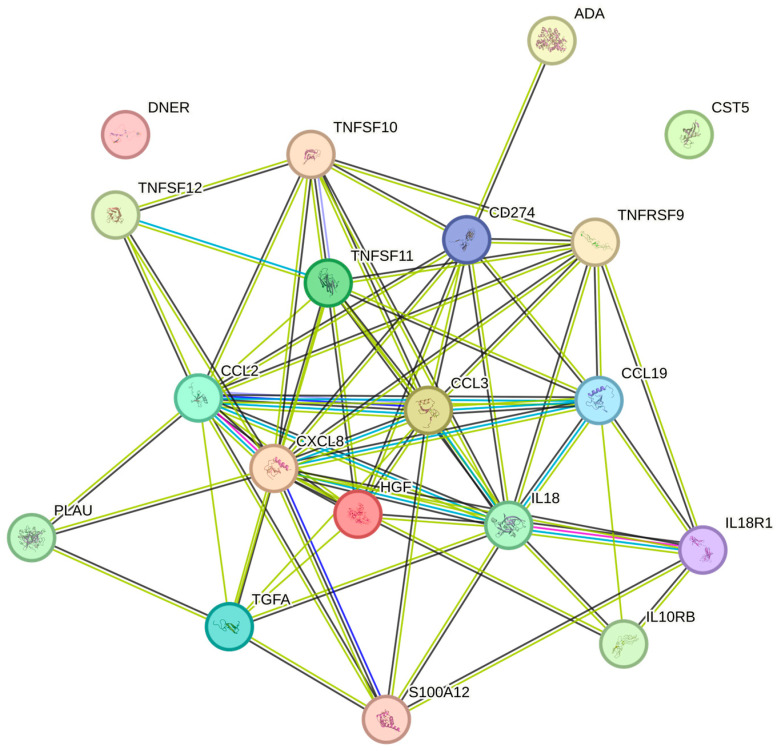
The figure represents a network of interactions among cytokines, demonstrating significant associations between circulating cytokine levels and the erythrocyte volume fraction. The nodes (cytokines) are connected by edges of varying thickness, indicating interaction strength.

The erythrocyte volume fraction was significantly associated with TRAIL, MCP-1, HGF, TNFRSF9, ADA, DNER, CCL19, IL-10RB, IL18, TWEAK, CST5, PD-L1, CCL3, uPA, EN-RAGE, TRANCE, IL-18R1, TGF-alpha, and IL8 (Table 2). The most expressed associations were noted between TRAIL (*p* = 0.001), MCP-1 (*p* = 0.002), and HGF (*p* = 0.006), respectively (Figure 5).

The hemoglobin values were significantly associated with TRAIL, MCP-1, HGF, ADA, DNER, TNFRSF9, IL18, uPA, EN-RAGE, TWEAK, and TRANCE (Table 2).

There were also significant associations between MCHC and IL-12B and between MCV and IL-18R1.

## 3. Discussion

All studied PEA markers in this comparison have previously been proposed as either markers of inflammation or cardiovascular risk or both. The PEA assay unites high specificity, sensitivity, and low sample consumption and is therefore well suited for the detection of protein biomarkers [32]. We found several significant associations between blood cell parameters and cytokine levels, not only for the white blood cells but also for red blood cells and platelets. We also showed that within each cell type, there were significant associations between different cytokines, even when they did not belong to the same cytokine family (e.g., erythrocytes were associated with TNFSF9/10/11/12, IL-18, and CCL2), which were interrelated. It is noteworthy that the ten highest correlations between cell types and associated cytokine levels also had the lowest significance levels. There was no particular cell type, cytokine, or even cytokine family that constituted any link between these cells or cytokines. Since the most significant association was noted between the neutrophil count and OSM, followed by white blood cells and OSM, it is tempting to speculate that the main effect of white blood cells on OSM refers to neutrophils. We also noted associations, in decreasing order, between the number of white blood cells, neutrophil count, platelet count, and IL-6. We did not find any association between the platelet count and OSM, even though this cytokine belongs to the same family as IL-6 [33]. TRAIL, one of the cytokines with high correlation and low significance level, belonging to the TNF family [34], was, in decreasing order, significantly correlated to the erythrocyte volume fraction, hemoglobin, erythrocyte count, and, to some extent, also to the platelet count.

Our findings align with prior research. Several of the cytokines studied showed associations with platelet counts. FGF-23 has previously been shown to have a positive association with mean platelet volume [35]. Patients treated with rhuIL-10 developed decreased hemoglobin values and platelet counts [36]. Platelets were a major source of TGF-beta1 in the blood [37]. Regulation of protein acetylation by SIRT2 had an important role for platelet function [38]. IL-6 was shown to stimulate megakaryocytes to increase platelet production [39]. The interaction between CD40 and CD40L contributed to the binding of platelets to white blood cells [40]. Thus, there were several links between the platelet counts and the significantly associated cytokines in this study.

Cytokine–blood cell interactions have played a crucial role in developing therapeutic applications for various diseases, particularly in immunology, oncology, and inflammatory conditions. Some examples of these therapeutic applications are: Recombinant human erythropoietin (EPO) is used to treat anemia associated with chronic kidney disease and has improved the quality of life for kidney dialysis patients. G-CSF analogs (filgrastim) are used to treat neutropenia, especially in chemotherapy-induced neutropenia and congenital neutropenia, reducing the risk of infections. IL-6 inhibitors (tocilizumab, sarilumab) are used to treat rheumatoid arthritis, giant cell arteritis, and cytokine release syndrome from CAR-T cell therapy. TNF inhibitors (infliximab, adalimumab, etanercept) are used in treating autoimmune diseases like rheumatoid arthritis, Crohn’s disease, ulcerative colitis, psoriasis, and ankylosing spondylitis [41,42,43,44].

### Limitations

This study has several limitations. Firstly, it was a single-site study. Secondly, due to geographic factors, the majority of the patients were Caucasians. Thirdly, those who were recruited were within a limited age span. Furthermore, the patients were not subjected to laboratory screening for co-existing diseases; instead, they were assessed clinically and deemed to be in good health. Thus, we cannot deduce to what extent, if any, either acute or chronic diseases may affect the associations between blood cell parameters, cytokines, chemokines, and growth factors. No protease inhibitor was used during sample collection, which means some degree of proteolysis may have occurred in the samples.

## 4. Materials and Methods

### 4.1. Population

This study included 165 essentially healthy patients, aged 18 to 44 years, weighing between 50 and 115 kg. All participants were previously referred to the Oral and Maxillofacial Surgery Clinic at Falun County Hospital, Sweden, for the surgical removal of an impacted lower third molar. Subjects with well-compensated systemic disease were accepted for inclusion in this study. The exclusion criteria were described in a previous publication [45].

### 4.2. Sampling Procedures

Blood samples were collected in vacutainer tubes while the patient was in the supine position. A peripheral venous catheter was used for sample collection. Complete blood cell counts were analyzed at the routine laboratory of Falun Hospital, Sweden. All blood samples were obtained prior to surgery. Plasma cytokine samples (EDTA) were frozen and stored at −80 °C until analysis.

### 4.3. Ethics

This study was conducted in accordance with the principles of the Helsinki Declaration [46]. It received approval from the Swedish Ethical Review Authority (Dnr 2015/378) and was registered in the European Union Regulating Authorities Clinical Trials Database (EudraCT) under number 2014-004235-39. Additionally, it is listed on ClinicalTrials.gov with the ID NCT04459377. All participants provided both oral and written informed consent before taking part in the study.

### 4.4. Proximity Extension Assay (PEA)

The proximity extension assay (PEA) was performed using the Proseek Multiplex Inflammation kit (Olink Bioscience, Uppsala, Sweden) [32]. In brief, 1 µL of plasma was combined with 3 µL of incubation mix, containing two probes (antibodies conjugated with unique DNA oligonucleotides). The mixture was incubated at 8 °C overnight.

Following incubation, 96 µL of extension mix, which included the PEA enzyme and PCR reagents, was added. The samples were then incubated at room temperature for 5 min before undergoing 17 cycles of DNA amplification in a thermal cycler.

A 96.96 Dynamic Array IFC (Fluidigm, South San Francisco, CA, USA) was prepared and primed according to the manufacturer’s guidelines. In a separate plate, 2.8 µL of the sample mixture was combined with 7.2 µL of detection mix, and 5 µL of this solution was loaded into the right side of the primed 96.96 Dynamic Array IFC. Unique primer pairs for each cytokine were loaded into the left side of the array. The protein expression analysis was then performed using the Fluidigm Biomark reader, following the Proseek protocol. Each Proseek kit quantified 92 biomarkers, resulting in a total analysis of 194 biomarkers in the study.

### 4.5. Statistical Analysis

Coefficients of variation were analyzed using Spearman rank correlations with Statistica (StatSoft (Version 14), Tulsa, OK, USA). Cytokine values that were above or below the highest and lowest standard points were assigned the values of these standards. To address the increased risk of false discoveries when analyzing a large number of correlations, *p*-values were adjusted for multiplicity using the false discovery rate (FDR) approach [47]. Adjusted *p*-values less than 0.10, corresponding to an expected false discovery rate of at most 10%, were considered significant. Results were adjusted for multiple testing, with a significance threshold of *p* < 0.05.

## 5. Conclusions

The observed associations between blood cell counts and circulating cytokines suggest potential applications in diagnostic and monitoring strategies for immune-mediated diseases and inflammatory conditions. For example, platelet count and IL-6, which showed strong associations in this study, have previously been linked to cardiovascular risk and inflammatory disorders [48]. Moreover, the identification of intercytokine interactions across different families may reflect coordinated inflammatory pathways that are relevant for disease progression, stratification, therapeutic response, and novel biomarker medicine approaches [49]. Future studies are warranted to validate these findings in disease cohorts and to explore their clinical utility in monitoring treatment response or predicting disease progression.

## Figures and Tables

**Table 1:** Number, weight (kg), and height (cm).

	Valid N	Geometric Mean	Minimum	Maximum	Std. Dev.	CV
Weight	165	73	48	111	13	17
Height	165	171	153	195	8.6	5
BMI	165	25	15	41	4.4	17
hsCRP	165	0.8	0.2	10	2.0	250
Hb	165	137	94	167	12.1	9
RBC	165	5	4	6	0.41	9
EVF	165	0.41	0.14	0.48	0.04	10
Platelets	165	238	100	430	52	21
WBC	165	5.7	3.4	13.1	1.8	31
Neutrophils	165	3.1	0.5	9.5	1.4	42

Abbreviations: Body mass index (BMI; kg × m^−2^), high sensitivity C-reactive protein (hsCRP; mg × L^−1^), hemoglobin (Hb; g × L^−1^), erythrocyte count (RBC; 10^12^ × L^−1^), erythrocyte volume fraction (EVF; %), platelet count (platelets; 10^9^ × L^−1^), white blood cell count (WBC; 10^9^ × L^−1^), and neutrophil count (neutrophils; 10^9^ × L^−1^). Standard deviation (Std. Dev.), CV (coefficients of variation).

**Table 2:** Cytokines, chemokines, and growth factors and their relations to cell types and red blood cell parameters, determined by both Benjamini–Hochberg *p*-values and Spearman rank correlations.

Blood Cell Marker	Cytokine	UniProt ID	N	Spearman Rank Correlations	Benjamini–Hochberg *p*-Value
EVF	TRAIL	P50591	165	0.349	0.001
EVF	MCP-1	P13500	165	0.321	0.002
EVF	HGF	P14210	165	0.304	0.006
EVF	TNFRSF9	Q07011	165	0.259	0.022
EVF	ADA	P00813	165	0.251	0.031
EVF	DNER	Q8NFT8	165	0.236	0.046
EVF	CCL19	Q99731	165	0.23	0.051
EVF	IL-10RB	Q08334	165	0.229	0.051
EVF	IL18	Q14116	165	0.228	0.051
EVF	TWEAK	O43508	165	0.224	0.053
EVF	CST5	P28325	165	0.217	0.061
EVF	PD-L1	Q9NZQ7	165	0.213	0.067
EVF	CCL3	P10147	165	0.212	0.069
EVF	uPA	P00749	165	0.211	0.069
EVF	EN-RAGE	P80511	165	0.205	0.076
EVF	TRANCE	O14788	165	0.205	0.076
EVF	IL-18R1	Q13478	165	0.202	0.081
EVF	TGF-alpha	P01135	165	0.198	0.088
EVF	IL8	P10145	165	0.196	0.092
Hb	TRAIL	P50591	165	0.337	0.001
Hb	MCP-1	P13500	165	0.291	0.011
Hb	HGF	P14210	165	0.276	0.017
Hb	ADA	P00813	165	0.233	0.049
Hb	DNER	Q8NFT8	165	0.232	0.049
Hb	TNFRSF9	Q07011	165	0.229	0.051
Hb	IL18	Q14116	165	0.205	0.076
Hb	uPA	P00749	165	0.200	0.084
Hb	EN-RAGE	P80511	165	0.199	0.087
Hb	TWEAK	O43508	165	0.196	0.092
Hb	TRANCE	O14788	165	0.193	0.099
MCHC	IL-12B	P29460	165	−0.214	0.066
MCV	IL-18R1	Q13478	165	−0.229	0.051
Neutroph	OSM	P13725	165	0.505	0
Neutroph	IL6	P05231	165	0.327	0.002
Neutroph	TNFSF14	O43557	165	0.241	0.040
Neutroph	HGF	P14210	165	0.232	0.049
Neutroph	EN-RAGE	P80511	165	0.214	0.067
Neutroph	SCF	P21583	165	−0.204	0.079
Plt	FGF-23	Q9GZV9	165	0.282	0.016
Plt	IL-10RB	Q08334	165	0.269	0.017
Plt	LAP TGF-beta-1	P01137	165	0.269	0.017
Plt	CCL19	Q99731	165	0.263	0.021
Plt	SIRT2	Q8IXJ6	165	0.261	0.022
Plt	IL6	P05231	165	0.26	0.022
Plt	VEGFA	P15692	165	0.257	0.024
Plt	AXIN1	O15169	165	0.244	0.037
Plt	CASP-8	Q14790	165	0.239	0.042
Plt	CD40	P25942	165	0.231	0.050
Plt	PD-L1	Q9NZQ7	165	0.227	0.051
Plt	CXCL5	P42830	165	0.227	0.051
Plt	MCP-3	P80098	165	0.225	0.052
Plt	STAMBP	O95630	165	0.225	0.052
Plt	CCL3	P10147	165	0.223	0.054
Plt	NRTN	Q99748	165	0.223	0.054
Plt	HGF	P14210	165	0.218	0.059
Plt	CD5	P06127	165	0.216	0.063
Plt	TRAIL	P50591	165	0.214	0.067
Plt	CCL4	P13236	165	0.210	0.070
Plt	ADA	P00813	165	0.210	0.071
Plt	TNFSF14	O43557	165	0.208	0.072
Plt	MCP-4	Q99616	165	0.193	0.099
RBC	TRAIL	P50591	165	0.277	0.017
RBC	IL-18R1	Q13478	165	0.270	0.017
RBC	HGF	P14210	165	0.270	0.017
RBC	MCP-1	P13500	165	0.240	0.042
RBC	TRANCE	O14788	165	0.227	0.051
RBC	TNFRSF9	Q07011	165	0.224	0.053
RBC	ADA	P00813	165	0.211	0.069
RBC	EN-RAGE	P80511	165	0.209	0.071
RBC	CASP-8	Q14790	165	0.199	0.086
WBC	OSM	P13725	165	0.463	0
WBC	IL6	P05231	165	0.325	0.002
WBC	HGF	P14210	165	0.277	0.017
WBC	TNFSF14	O43557	165	0.265	0.020
WBC	EN-RAGE	P80511	165	0.222	0.054
WBC	TGF-alpha	P01135	165	0.212	0.069
WBC	CCL19	Q99731	165	0.203	0.079
WBC	CCL11	P51671	165	−0.197	0.090

Abbreviations: erythrocyte count (RBC; 10^12^ × L^−1^), erythrocyte volume fraction (EVF; %), hemoglobin (Hb; g × L^−1^), mean corpuscular hemoglobin (MCHC; g × L^−1^), mean corpuscular volume (MCV; fl, 10^−15^), erythrocyte volume fraction white blood cell count (WBC; 10^9^ × L^−1^), neutrophil count (Neutroph; 10^9^ × L^−1^), and platelet count (Plt; 10^9^ × L^−1^).

## Data Availability

Under Swedish Law, the authors cannot share the data used in this study and cannot conduct any further research other than what is specified in the ethical permissions application. For inquiries about the data, researchers should first contact the owner of the database, the University of Uppsala, Sweden. Please contact the corresponding author with requests for and assistance with data. If the university approves the request, researchers can submit an application to the Regional Ethical Review Board for the specific research question that the researcher wants to examine.

## Supplementary Materials

The supporting information can be downloaded at: https://www.mdpi.com/article/10.3390/ijms26094065/s1.
