# Supplementary material for: Significant Associations Between Blood Cell Counts and Plasma Cytokines, Chemokines, and Growth Factors
Source: Int J Mol Sci. 2025 Apr 25;26(9):4065. doi: 10.3390/ijms26094065 (PMC12072032; doi:10.3390/ijms26094065)
Supplement: Supplementary file 1 [file ijms-26-04065-s001.zip › Table S1.pdf]

Supplementary Table S1.

UniProt ID	Gene	Protein name
P30203	CD6	T-cell differentiation antigen CD6
P21583	KITLG	Kit ligand
Q14116	IL18	Interleukin-18
Q13291	SLAMF1	Signaling lymphocytic activation molecule
P01135	TGFA	Protransforming growth factor alpha
Q99616	CCL13	C-C motif chemokine 13
P51671	CCL11	Eotaxin
O43557	TNFSF14	Tumor necrosis factor ligand superfamily member 14
Q9GZV9	FGF23	Fibroblast growth factor 23
Q13651	IL10RA	Interleukin-10 receptor subunit alpha
P13236	CCL4	C-C motif chemokine 4
P12034	FGF5	Fibroblast growth factor 5
P42702	LIFR	Leukemia inhibitory factor receptor
Q9NSA1	FGF21	Fibroblast growth factor 21
Q99731	CCL19	C-C motif chemokine 19
Q13261	IL15RA	Interleukin-15 receptor subunit alpha
Q08334	IL10RB	Interleukin-10 receptor subunit beta
Q8N6P7	IL22RA1	Interleukin-22 receptor subunit alpha-1
Q13478	IL18R1	Interleukin-18 receptor 1
Q9NZQ7	CD274	Programmed cell death 1 ligand 1
P01138	NGF	Beta-nerve growth factor
P42830	CXCL5	C-X-C motif chemokine 5
P03956	MMP1	Interstitial collagenase
O14788	TNFSF11	Tumor necrosis factor ligand superfamily member 11
Q969D9	TSLP	Thymic stromal lymphopoietin
P60568	IL2	Interleukin-2
P15692	VEGFA	Vascular endothelial growth factor A
P80098	CCL7	C-C motif chemokine 7
P39905	GDNF	Glial cell line-derived neurotrophic factor
Q9H5V8	CDCP1	CUB domain-containing protein 1
Q9BZW8	CD244	Natural killer cell receptor 2B4
P13232	IL7	Interleukin-7
O00300	TNFRSF11B	Tumor necrosis factor receptor superfamily member 11B
P01137	TGFB1	Transforming growth factor beta-1 proprotein
P00749	PLAU	Urokinase-type plasminogen activator
P05231	IL6	Interleukin-6
P09341	CXCL1	Growth-regulated alpha protein
Q9P0M4	IL17C	Interleukin-17C
Q16552	IL17A	Interleukin-17A

O14625	CXCL11	C-X-C motif chemokine 11
O15169	AXIN1	Axin-1
P50591	TNFSF10	Tumor necrosis factor ligand superfamily member 10
Q9UHF4	IL20RA	Interleukin-20 receptor subunit alpha
Q07325	CXCL9	C-X-C motif chemokine 9
P28325	CST5	Cystatin-D
P14784	IL2RB	Interleukin-2 receptor subunit beta
P01583	IL1A	Interleukin-1 alpha
P13725	OSM	Oncostatin-M
P13500	CCL2	C-C motif chemokine 2
P10145	CXCL8	Interleukin-8
Q8NFT8	DNER	Delta and Notch-like epidermal growth factor-related receptor
Q9NRJ3	CCL28	C-C motif chemokine 28
Q8IXJ6	SIRT2	NAD-dependent protein deacetylase sirtuin-2
Q9NYY1	IL20	Interleukin-20
Q13541	EIF4EBP1	Eukaryotic translation initiation factor 4E-binding protein 1
P02778	CXCL10	C-X-C motif chemokine 10
P80162	CXCL6	C-X-C motif chemokine 6
P49771	FLT3LG	Fms-related tyrosine kinase 3 ligand
P80511	S100A12	Protein S100-A12
P10147	CCL3	C-C motif chemokine 3
P55773	CCL23	C-C motif chemokine 23
P01375	TNF	Tumor necrosis factor
P22301	IL10	Interleukin-10
P09238	MMP10	Stromelysin-2
Q5T4W7	ARTN	Artemin
P35225	IL13	Interleukin-13
Q13007	IL24	Interleukin-24
P29460	IL12B	Interleukin-12 subunit beta
P06127	CD5	T-cell surface glycoprotein CD5
P14210	HGF	Hepatocyte growth factor
P25942	CD40	Tumor necrosis factor receptor superfamily member 5
P01579	IFNG	Interferon gamma
P09603	CSF1	Macrophage colony-stimulating factor 1
P01374	LTA	Lymphotoxin-alpha
P00813	ADA	Adenosine deaminase
P05113	IL5	Interleukin-5
O95630	STAMBP	STAM-binding protein
P50225	SULT1A1	Sulfotransferase 1A1
P78556	CCL20	C-C motif chemokine 20
O43508	TNFSF12	Tumor necrosis factor ligand superfamily member 12

O95760	IL33	Interleukin-33
P20783	NTF3	Neurotrophin-3
P78423	CX3CL1	Fractalkine
O15444	CCL25	C-C motif chemokine 25
Q14790	CASP8	Caspase-8
P80075	CCL8	C-C motif chemokine 8
Q99748	NRTN	Neurturin
P15018	LIF	Leukemia inhibitory factor
P05112	IL4	Interleukin-4
O95750	FGF19	Fibroblast growth factor 19
Q07011	TNFRSF9	Tumor necrosis factor receptor superfamily member 9
P01732	CD8A	T-cell surface glycoprotein CD8 alpha chain

Supplementary Table S1. List of plasma cytokines, chemokines, and growth factors analyzed, using the Proseek Multiplex Inflammation kit (Olink Bioscience, Uppsala, Sweden).
